# Mississippi State Medical Association

**Published:** 1881-10-20

**Authors:** 


### *MISSISSIPPI STATE MEDICAL ASSOCIATION.*

We are in receipt of a copy of the transactions of the Mississippi State Medical Association, at the fourteenth annual session held at Winona, April 6th, 7th and 8th, 1881, an interesting and well gotten up work of 196 pages. We have space only to name the following papers: President's Address, by Dr. W. F. Hyer; Rights, Duties and Responsibilities of Physicians before the courts, by Hon. J. S. Morris; Abortive Treatment of Pneumonia, by Dr. W. Y. Gadbury; New Remedies, by Dr. B. A. Vaughan; Diphtheria, by Dr. J. B. Gresham; Recent Advances in General Pathology, by Dr. B. F. Ward; Diseases of the Gastro-Enteric Mucous Membrane in Infancy and Childhood, by Dr. R. F. Ward.

*Report on the Surgery of Mississippi*—By Dr. M. S. Craft.

Cases reported by Dr. Brownrigg, of Columbus; Dr. R. A. Cunningham, of Verona; Dr. R. R. Blailock, of Carthage; Dr. J. H. Blanks, of Meridian; Dr. W. D. Carter, of Ripley; Dr. S. V. D. Hill, of Macon; Dr. F. E. Daniel, of Jackson; Dr. J. M. Greene, of Aberdeen; Dr. B. F. Kittrell, of Black Hawk· Dr. L. M. Mays, of Graysport; Dr. A. P. Harris, of Edwards; Dr. M. S. Craft, of Jackson; New Appliances for Fracture of Lower Extremities, by Dr. W. Y. Gadbury; Report of Committee on Necrology, Dr. A. G. Smythe, chairman.

### OFFICERS ELECT.

*President*—B. F. Ward, M. D., Winona.

*Vice-Presidents*—1st. J. P. Moore, M. D., Yazoo City. 2d. T. W. Fullilove, M. D., Vaiden. 3d. John Tackett, M. D., Richland. 4th. W. W. Hart, M. D., Lodi.

*Recording Secretary*—Wirt Johnston, M. D., Jackson.
*Corresponding Secretary*—M. S. Craft, M. D., Jackson.
*Treasurer*—G. K. Harrington, M. D., Jackson.
*Orator*—F. E. Daniel, M. D., Jackson.
*Alternate Orator*—T. R. Henderson, M. D., Greenwood.

The next meeting is appointed for Oxford, on the first Wednesday in April, 1882.

